# Significance of Vascular Endothelial Growth Factor Expression in the Bladder Urothelial Carcinoma and Its Association with Tumor Grade and Invasiveness

**DOI:** 10.30699/IJP.20201.138671.2518

**Published:** 2021-07-06

**Authors:** Anika Sadaf, Md. Zillur Rahman, Pradip Bhattacharjee, M. Shahab Uddin Ahamad, Sayeeda Nasreen

**Affiliations:** 1 *Department of Pathology, Chittagong Medical College, Chattogram, Bangladesh.*; 2 *Department of Pathology, Bangabandhu Sheikh Mujib Medical University, Dhaka, Bangladesh.*

**Keywords:** Angiogenesis, Bevacizumab, Muscle-invasion, Vascular endothelial growth factor, Urothelial carcinoma

## Abstract

**Background & Objective:**

Vascular Endothelial Growth Factor (VEGF) is one of the newer molecular markers that acts as a central mediator of tumor angiogenesis and is essential for tumor growth, progression, and metastasis. So anti-angiogenic drugs can be used as anticancer therapy. Treatments with anti-VEGF (Bevacizumab) therapy have been proved to improve relapse-free survival in many tumors. Urinary bladder tumor has become emerging cancer globally among elderly individuals. So, the identification and development of novel biomarkers for effective treatment of urinary bladder carcinoma is essential. The present study aimed to investigate the immunohistochemical expression of VEGF in urothelial carcinoma of urinary bladder and to assess its association with tumor grade and muscle invasiveness.

**Methods:**

This cross-sectional study was conducted in the Department of Pathology, Chittagong Medical College, Chattogram from September 2018 to August 2020. Fifty-six formalin-fixed paraffin-embedded tissue blocks of urinary bladder carcinoma were prepared for both histopathological and immunohistochemical examination. Each slide was evaluated by at least two pathologists.

**Results:**

Weak to strong positive expression of VEGF were observed in 52 cases (92.86%). The proportion of tumors positive for VEGF expression was higher among patients with high grade and non-muscle invasive bladder carcinoma.

**Conclusion:**

We found that VEGF expression has a significant association with tumor grade and an inverse association with muscle invasion. These findings may be useful for selecting the subset of patients likely to respond to anti-VEGF targeted therapy.

## Introduction

Carcinoma of the urinary bladder is the 10^th^ most common form of cancer worldwide. It is more prevalent in male than in female. The incidence of urinary bladder malignancies in men is 9.6 per 100,000 and the mortality rate is 3.2 per 100,000 worldwide (1). American Cancer Society estimated 60,490 new cases of urinary bladder carcinomas in men and 12,240 deaths among them in the United States in 2017 from population-based data (2). Though the prevalence is higher in developed countries, but incidence is gradually increasing in developing countries like Bangladesh, India etc. due to raising occupational exposure and smoking habit (3).

Bladder cancer generally originates from urothe-lium and urothelial carcinomas (UC) are the most common type. Approximately 75% of newly diagnosed patients have non-muscle-invasive bladder cancer (NMIBC) (stage- PTa/ PTis/ PT1) and 25% have MIBC or metastatic disease (stage- PT2-PT4) (4). Despite intravesical treatments, 50%-70% patients with NMIBC recur within 5 years and up to 30% progress to MIBC (5). The literature demonstrates the therapeutic and prognostic value of biomarkers involved in the bio-molecular mechanism of urothelial carcinoma and other urinary tract tumors like CXR2 and CXR3 in renal cell carcinoma and prostate-specific antigen (PSA) in prostatic adenocarcinoma (6-11).

Angiogenesis is essential to support the growth of solid tumors. Vascular endothelial growth factor (VEGF) is one of the essential growth factors involved in vasculogenesis and angiogenesis. It is produced by a variety of normal and neoplastic cells and production is regulated by hypoxia. VEGF level varies from high to low in hypoxic to normoxic regions of the tumor. VEGF binds to its cognate receptors, VEGFR1 and VEGFR2 and induce cell proliferation and migration (12). It can anticipate the rates of tumor growth and disease outcome to correlate with the abundance of VEGF level. There are evidence to support the existence of VEGF in several cancer types including bladder cancer. In addition, by increasing vascular permeability, VEGF facilitates the entry of tumor cells into circulation and allows them to metastasize to distant sites (13). Many tumor cells and tumor associated vasculature express both VEGF and VEGFRs. The expression of VEGF and its receptors on Kaposi sarcoma, melanoma, ovarian carcinoma, squamous cell carcinoma of the head and neck and breast carcinoma have been reported in last several years (12).

Anti-angiogenic drugs, mainly Bevacizumab, Sora-fenib and Sunitinib are already approved for use in many advanced tumors, such as breast, colorectal, liver and kidney cancer; and they are seen to significantly improve the treatment of cancer (7, 8, 14). Several studies have suggested that inhibition of VEGF transcripts significantly reduced the proliferation rate of the bladder cancer cells (15) and some studies have shown that blockade of VEGF receptor reduced growth and invasion of bladder cancer cells (16,17). Treatment of advanced UC with Bevacizumab (VEGF antibody) and Ramucirumab (VEGFR2 antibody), in combi-nation with chemotherapy showed promising results in phase II clinical trials. A current phase III trial compares Gemcitabine/ Cisplatin (GC) chemotherapy with GC plus Bevacizumab for metastatic or unresectable UC. There are multiple ongoing phase II studies with other agents targeting VEGF receptors, including Sunitinib, Sorafenib and Pazopanib (18, 19).

The present study was conducted to observe the expression of VEGF in paraffin embedded tissues taken from histologically confirmed urothelial carci-noma and its association with tumor grade and muscle-invasiveness. So far known, there was no such previous study in Bangladesh. This could be helpful for better understanding regarding the prognostic value of biomarker and the role of anti-VEGF drugs for the effective treatment of urothelial carcinoma.

## Material and Methods

This was a cross sectional observational study conducted in Department of Pathology, Chittagong Medical College, Chattogram, Bangladesh, from September 2018 to August 2020. The authors had prior approval from Institutional Review Board (IRB). Sixty-one consecutive cases of urinary bladder carcinoma were histopathologically examined. Patients other than primary urothelial carcinoma or patients previously treated with chemotherapy or radiotherapy for bladder tumor were excluded. Finally, 56 cases of urothelial carcinomas were prepared for immuno-histochemical examination. Data was recorded considering the variables of interest by structured interview and then documented in a pre-designed case record form after taking informed written consent from the patients. Socio-economic profile of the patients was classified according to *modified Kuppus-wamy Socioeconomic status* scale (20). Patients were eligible for inclusion if they were undergone cystoscopy involved collecting a biopsy or transurethral resection of bladder tumor (TURBT) and all the specimens of each case were submitted for histological examination; and finally diagnosed as primary urothelial carcinoma according to the WHO classification. Tumor grade was evaluated according to 2004 WHO grading system of urothelial tumor. Invasiveness was recorded depending on invasion of the muscularis propria (detrusor muscle) by malignant cells.

Immunohistochemical Evaluation

The most representative tumor tissue was chosen from each case and 3-5 μm thickness section of formalin fixed paraffin embedded tissues was taken on poly-L-lysine coated slide. After de-paraffinization and rehydration, antigen retrieval was done by microwave at 750°C for 15 min. Then the sections were stained with primary antibody against VEGF (monoclonal, dilution 1:50, INVITROGEN, ThermoFisher Scient-ific, USA) for 30 min. Staining was done using sub-stratechromogen solution: DAB and counterstain was done by H & E staining. VEGF status was scored based on intensity of reactivity and the percentage of reactive cells. Cytoplasm and/or membrane staining was considered as immune-positive. The scoring was done by the 40x objective lens and counting at least 100 cells for immunoreactivity in 10 fields. The results of staining intensity (0-no stain/ colorless, 1-slight staining/ yellowish, 2- moderate staining/ brown-yellow, 3-maximal staining/dark brown) and the percentage of positive cells (0- no stain, 1- <25% positive cells, 2- 25-50% positive cells, 3->50% positive cells) were added and the final score of immunoexpression for VEGF was recorded as 0: negative ( - ), 1-2: weak positive ( + ), 3-4: moderate positive ( + + ), 5-6: strong positive (+ + +). Renal tubules were used as external control. Omission of the primary antibody was used for negative control.

Statistical Analysis

SPSS 25 (SPSS Inc., Chicago, Ill., USA) was used for data analysis. Fisher exact test and ANOVA test were performed to find out the possible association of VEGF expression with different variables. P-value< 0.05 was considered statistically significant and confidence interval was set at 95% level.

## Results

A total of 56 histologically confirmed cases of urothelial carcinomas comprising 48 (85.7%) males and 8 females were assessed from the TURBT samples. The mean age of the patients was 60.96 ± 12.93 years, ranging from 28 to 95 years; male to female ratio was 6:1. Most of the patients (47 cases; 83.9%) were involved in non-industrial works. Rests were industrial workers; among them, there were 2 leather workers, 2 machinery workers, 2 painters, 2 fertilizer workers and 1 soap-factory worker. All cases were belonged from low and middle socio-economic class of *modified Kuppuswamy Socioeconomic status* scale (20). Distribution of the patients according to smoking habits is shown in Table 1.

Regarding past history, six patients (10.7%) had history of chronic cystitis, 3 patients (5.4%) were taking immunosuppressive drugs (2 for arthritis and 1 for SLE), 2 patients (3.5%) had history of instrument-tation for TURP and one had history of both chronic cystitis and immunosuppressive drugs for arthritis.

The site of tumor was assessed through ultrasonography or CT scan. Twenty-two of the tumors (39.3%) were found on the right or left lateral wall of the urinary bladder. Rests were on posterior wall (5 cases; 8.9%), anterior wall (4 cases; 7.1%), base (3 cases; 5.4%), multiple sites (17 cases; 30.4%) and as thicken bladder wall (5 cases; 8.9%).

On microscopic evaluation, high grade (31 cases; 55.4%) and MIBC (40 cases; 71.43%) cases showed predominance. The relationship between different histological grades and muscle invasiveness was found statistically significant (*P*<0.05). Statistically significant associations (*P*<0.05) were also evident among VEGF expressions with tumor grade and muscle-invasiveness. Findings obtained from histopa-thological and immunohistochemical examinations are assembled in Tables 2, 3 and 4**.**

**Table 1:** Distribution of the patients according to smoking habit (n=56)

	Frequency (%)
Smoking habit	
Tobacco smoker	42 (75.0)
Non-tobacco smoker	14 (25.0)
Duration of smoking (Years) (n=42)	
<10	2 (04.76)
10-20	16 (38.10)
21-30	12 (28.57)
>30	12 (28.57)

**Fig 1:**
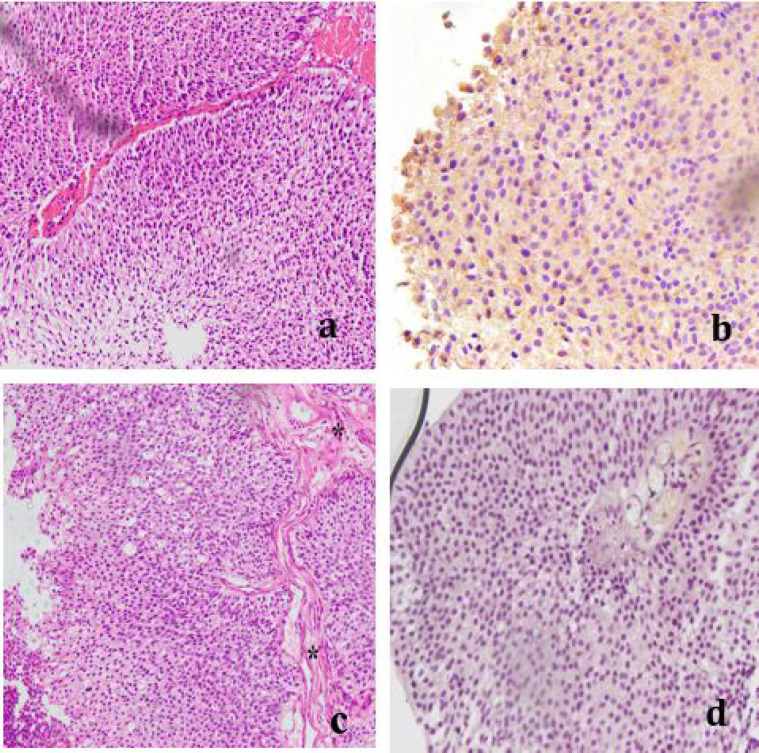
Urothelial carcinoma, Low grade **a**. NMIBC, H & E (x20); **b**. Strong positive (+++) VEGF expression (x40); **c.** MIBC, H & E (x20); **d**. Negative (-) VEGF expression (x40)

**Table 2:** Histopathological spectrum of bladder carcinoma (n=56)

	Frequency (%)
Histopathological grade	
High	31 (55.4)
Low	25 (44.6)
Muscle invasiveness	
MIBC	40 (71.43)
NMIBC	9 (16.07)
Muscle could not be identified	7 (12.50)

MIBC: Muscle invasive bladder carcinoma, NMIBC: Non-muscle invasive bladder carcinoma

**Table 3:** Relationship between histological grade and muscle invasiveness (n=49)

Muscle invasiveness	Histopathological grade		Total	P-value*
	Low	High		
NMIBC	09 (40.9)	00 (0.0)	09	**0.0002** ^s^
MIBC	13 (59.1)	27 (100.0)	40	
Total	22 (100.0)	27 (100.0)	49^Ψ^	

*Fisher exact test was done to measure the level of significance.

^Ψ^Seven cases were excluded as muscle could not be identified

**Table 4:** Expression of VEGF in among grades and muscle-invasiveness of bladder carcinoma

	VEGF expression				P-value*
	Negative (-)	Weak positive (+)	Moderate positive (++)	Strong positive (+++)	
Tumor grade (n= 56)					
Low	4 (16.0)	04 (16.0)	12 (48.0)	5 (20.0)	0.006^s^
High	0 (0.0)	12 (38.71)	18 (58.06)	1 (3.23)	
					
Muscle invasiveness (n=49)					
NMIBC	0 (0.0)	02 (22.22)	02 (22.22)	5 (55.56)	0.001^s^
MIBC	3 (7.5)	13 (32.5)	23 (57.5)	1 (2.5)	

*Fisher exact test was done to measure the level of significance. Figure within parenthesis indicates in percentage; s=statistically significant

**Table 5:** Comparison of VEGF expression in UBC among different studies

Author	Country	Total number of cases	VEGF expression	
			Positive	Negative
Yang *et al*. (27) 2004	Taiwan	161	54.70%	45.30%
Xia *et al*. (12) 2006	California, USA	72	97.20%	2.80%
Rahmani *et al*. (28) 2012	New Delhi, India	125	43.20%	56.80%
Kopparapu *et al*. (29) 2013	Philadelphia, USA	212	79.20%	20.80%
Adelmann *et al*. (30) 2018	Romania	50	13.33%	86.67%
Present study 2020	Bangladesh	56	92.86%	07.14%

**Fig 2:**
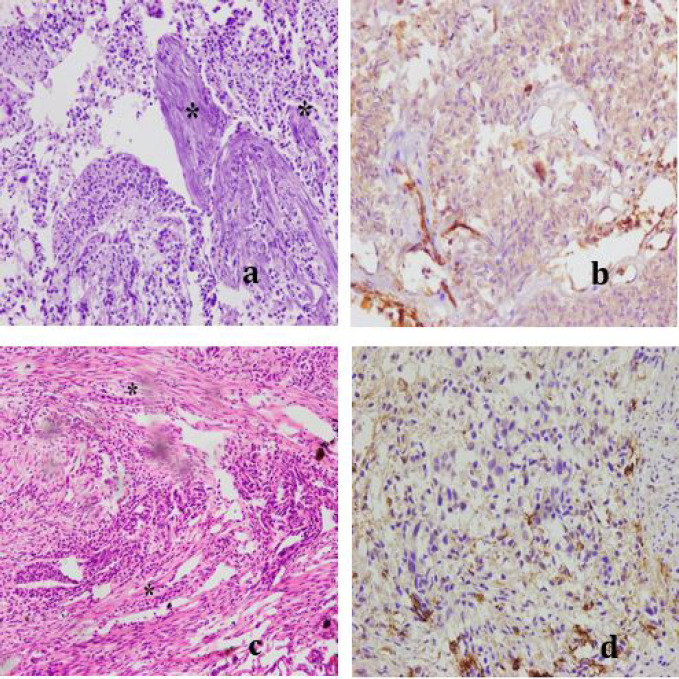
Urothelial carcinoma, High grade. **a.** MIBC, H & E (x20); **b.** Moderate positive (++) VEGF expression (x40); **c.** MIBC, H & E (x20); **d.** Weak positive (+) VEGF expression (x40) Asterisk (*) marks in MIBC cases indicate invasion of muscularis propria by malignant cells

## Discussion

Present study showed male predominance (48 cases; 85.74%) with 6:1 ratio. Gupta *et al. *in 2009, found a male to female ratio of 8.6:1 (21). The higher incidence of urinary bladder carcinoma in male may be due to the personal habit such as smoking and more exposure to carcinogenic agents due to their occupation. Rushton *et al. *in 2010 described that 5.6% urinary bladder malignancies were attributable to occupational exposure in their study (22).

We observed that, the incidence of high-grade urothelial carcinoma (HGUC) was higher in this study. Haque *et al*. (2018) recorded 72.0% patients with HGUC and remaining with low grade urothelial carcinoma (LGUC) (23). The study conducted by Chinnasamy *et al*. (2016) in India observed 63.4% cases of high-grade carcinoma and by the one performed by Chou *et al*. (2013) in Taiwan found 56.8% cases of high-grade carcinoma (24, 25). All these studies were in accordance with present study.

Current study found 40 cases (71.43%) with MIBC and 9 cases (16.07%) with NMIBC; while in rest of the cases (7 cases; 12.50%) muscularis propria could not be identified. Chou *et al*. in 2013 also found high incidence of MIBC; revealing 40.5% NMIBC and 59.5% MIBC (25). On the other hand, Chinnasamy *et al*. in 2016 recorded 86.5% cases of NMIBC (stage- PT1) and only 13.5% cases of MIBC (stage- PT2) (24). According to Thapa *et al.* in 2017 muscle invasion was observed in 24.45% cases of high-grade urothelial carcinomas; however, none of the cases of low grade papillary urothelial carcinoma showed muscle invasion (26). In current study, among twenty-two low grade carcinoma cases (n=49), 9 cases (40.9%) were NMIBC and 13 cases (59.1%) were MIBC. On the other hand, among 27 cases of high-grade carcinomas (n=49), all were MIBC. These differences between different tumor grades and muscle invasiveness were found statistically significant (*P*<0.05). Incidence of MIBC was high in our study. Poor socio-economic condition, lack of awareness, inadequate facilities of routine check-up for elderly people as well as social and religious restrictions in our country predispose to delayed healthcare seeking and may be the probable causes of increased rate of MIBC.

Our primary aim was to observe the immunohisto-chemical expression of VEGF in urinary bladder carcinoma. Table 5 shows the percentage of VEGF expression in urinary bladder carcinomas observed in different studies conducted in different countries. 

All the cases of high-grade carcinomas (31 cases) showed weak (+) to strong (+++) positive VEGF expression and none showed negative (-) expression. On the other hand, proportion of negative (-) VEGF expression was significantly higher among patients with low grade carcinoma. This indicated that with the progression in tumor grade, the rate of VEGF expression significantly increased (*P*<0.05); which was similar to Yang *et al*. (2004) and Rahmani *et al*. (2012) (27, 28). 

This study also evaluated the association between VEGF expression and muscle-invasiveness of urinary bladder carcinomas and revealed that all NMIBC cases were positive for VEGF expression and 55.56% (5 cases) showed strong positive (+++) expression. Whereas only 2.5% (1 case) of MIBC cases showed strong positive (+++) VEGF expression and 7.5% (3 cases) were negative (-) for VEGF expression. Statistically significant (*P*<0.05) differences were evident between VEGF expressions and muscle-invasiveness. Kopparapu *et al*. (2013) showed that VEGF expression was significantly higher in NMIBC compared to MIBC. The rate of tumor growth is higher at the early stage of the disease, which may be anticipated by VEGF. Thus, NMIBC may be correlated with the abundance of VEGF (29). Yang *et al*. in 2004 observed that the rate of positive expression of VEGF was significantly high in MIBC and furthermore, was associated with poor prognosis as seen in a 5-year follow up. So, they recommended VEGF as a useful prognostic marker for TCC (27). Özveren and Türkeri in 2019 observed that, the proportion of tumors positive for VEGF expression was higher in patients with NMIBC (45% vs 31%) and in high grade tumors (44% vs 37.5%). But statistically significant difference was not found in VEGF mRNA positivity with muscle-invasiveness by RT-PCR analysis of fresh tumor tissues (31). 

## Limitation

Information regarding extra-vesicular extension, lymph node and distal metastasis could not be included due to lack of logistic support, which could give more conclusive decision regarding prognostic significance of VEGF. 

## Conclusion

The data obtained in the present study led us to conclude that the proportion of tumors positive for VEGF expression was higher among patients with high grade and non-muscle invasive bladder carcinoma. The expression profile of VEGF may be useful for selecting high risk patients and predicting the subset of patients likely to response to anti- VEGF targeted therapy.
